# PemBla: A Phase 1 study of intravesical pembrolizumab in recurrent non‐muscle‐invasive bladder cancer

**DOI:** 10.1002/bco2.220

**Published:** 2023-01-13

**Authors:** Victoria K. Woodcock, Ji‐Li Chen, Karin Purshouse, Chrissie Butcher, Linda Collins, Caroline Haddon, Gillian Verrall, Leena Elhussein, Corran Roberts, Andrea Tarlton, Margarida Rei, Giorgio Napolitani, Mariolina Salio, Mark R. Middleton, Vincenzo Cerundolo, Jeremy Crew, Andrew S. Protheroe

**Affiliations:** ^1^ Department of Oncology Churchill Hospital, University of Oxford Oxford UK; ^2^ MRC Human Immunology Unit MRC Weatherall Institute of Molecular Medicine Oxford UK; ^3^ Oncology Clinical Trials Office, Department of Oncology University of Oxford Oxford UK; ^4^ Department of Urology Churchill Hospital Oxford UK; ^5^ Centre for Statistics in Medicine University of Oxford Oxford UK; ^6^ National Institute for Health Research Oxford Biomedical Research Centre Oxford UK

**Keywords:** bladder cancer, checkpoint inhibition, immunotherapy, intravesical, Phase 1

## Abstract

**Objectives:**

This study aimed to investigate the anti‐PD‐1 inhibitor pembrolizumab as a potential agent for use in non‐muscle‐invasive bladder cancer (NMIBC) by conducting a Phase 1 safety run‐in study to assess the safety and tolerability of intravesical pembrolizumab after transurethral resection of the bladder tumour (TURBT).

**Patients and methods:**

Eligible patients had recurrent NMIBC for which adjuvant treatment post TURBT was a reasonable treatment option, Eastern Cooperative Oncology Group Performance Status (ECOG PS) 0–1 and adequate end‐organ function. Pembrolizumab was administered by intravesical instillation once weekly for a total of six doses. Intra‐patient dose escalation was performed in three paired patient cohorts with doses starting at 50 mg and increasing through 100 mg to a maximum of 200 mg. Adverse events (AEs) were assessed using Common Terminology Criteria for Adverse Events (CTCAE) v4.03 with dose limiting toxicity (DLT) defined as a clinically significant, drug‐related, Grade 4 haematological or Grade 3 or higher non‐haematological toxicity occurring within 7 days of administration of the first treatment at a given dose for that patient.

**Results:**

Six patients were treated with no DLTs seen during dose escalation. Drug‐related AEs were of low grade and included dysuria and fatigue. All patients completed six doses of treatment as planned. Pharmacokinetic and pharmacodynamic assays did not detect any pembrolizumab in the serum following repeated intravesical administration, and no changes in peripheral immune cell populations were observed.

**Conclusions:**

Administration of intravesical pembrolizumab was well tolerated and did not raise any safety concerns in patients with NMIBC following TURBT. There was no evidence of systemic absorption or systemic immune effects following intravesical administration. Further studies are required to assess whether intravesical administration has anti‐tumour activity.

## 1 INTRODUCTION

Around half a million people worldwide are diagnosed with bladder cancer every year,^1^ with the majority of patients (75%–80%) diagnosed with non‐muscle‐invasive bladder cancer (NMIBC).^2^ Initial treatment of NMIBC consists of transurethral resection of the bladder tumour (TURBT). Instillation of a single dose of intravesical chemotherapy at the time of initial TURBT has been shown in meta‐analyses to significantly reduce recurrence rates^3^, ^4^, ^5^ and, in practice, mitomycin C (MMC) or gemcitabine are commonly used. Subsequent management is guided by risk stratification based on the pathology results of the resected tumour. Patients can broadly be grouped into low, intermediate or high risk according to the European Association of Urologists (EAU) guidelines,^6^ with individual risks of recurrence and progression being established using tables developed by the European Organisation for Research and Treatment of Cancer (EORTC).^7^ Further instillation of chemotherapy or immunotherapy (typically Bacillus Calmette‐Guerin [BCG]) into the bladder may be performed for patients at intermediate or high risk. Despite adjuvant treatment, bladder cancer has a propensity to recur and repeated resection of recurrences and further intravesical chemotherapy or BCG, or in some cases cystectomy, may be required.

BCG is the mainstay of treatment for intermediate‐ and high‐risk NMIBC and is thought to induce an influx of inflammatory cells driven by cytokines such as tumour necrosis factor‐alpha (TNF‐α), resulting in an immune response against tumour cells.^8^ However, BCG treatment failure rates resulting in progression or recurrence can be as high as 40%.^9^ Further, side effects such as cystitis and fevers can delay or terminate treatment in 20%–30% of patients.^9^, ^10^

In recent years, monoclonal antibodies blocking immune checkpoints have become established as standard of care in a range of cancers. Binding of PD‐L1 expressed on tumours, including some urothelial cancers,^11^ to PD‐1 on effector T cells attenuates their function and inhibits the anti‐tumour immune response.^12^ A number of treatments targeting the PD‐1/PD‐L1 axis have demonstrated activity in and gained approval for treatment of urothelial cancer in both the metastatic setting, for chemotherapy‐resistant^13^, ^14^, ^15^, ^16^, ^17^ or ‐ineligible patients,^18^, ^19^ and for patients with high‐risk NMIBC who are BCG unresponsive and not eligible for or have opted not to undergo cystectomy.^20^ The durable anti‐tumour responses generated by anti‐PD‐1 treatments and acceptable toxicity profile in these settings raise the possibility of new approaches to treatment for NMIBC. In particular, targeted administration via intravesical injection could potentially improve delivery of anti‐PD‐1 to exhausted T cells within the tumour site, while reducing toxicity due to systemic administration. Previous studies of intravesical injection of radiolabelled antibodies directed against tumour‐associated antigens have successfully showed retention of antibodies within the tumour mass,^21^ suggesting that this could be a feasible route for delivery of anti‐PD‐1 antibodies in the setting of NMIBC.

We sought to investigate the anti‐PD‐1 inhibitor pembrolizumab as a potential adjuvant intravesical agent for use in patients with intermediate‐risk NMIBC. Here, we discuss the results of the Phase 1 safety run‐in study (PemBla), the primary objective of which was to assess the safety, tolerability and toxicities of intravesical administration of pembrolizumab after TURBT. Exploratory objectives included investigating the pharmacokinetics and pharmacodynamics of intravesical pembrolizumab administration and evaluating the effects on the systemic immune response.

## 2 PATIENTS AND METHODS

### 2.1 Population

Patients were eligible if they were aged 18 years or over with recurrent NMIBC for which adjuvant treatment post TURBT was considered reasonable. Additional inclusion criteria included an Eastern Cooperative Oncology Group (ECOG) Performance Status 0 or 1, normal upper urinary tract with no evidence of tumour in the prostatic urethra at flexible cystoscopy and the following haematological and biochemical indices: haemoglobin ≥ 9.0 g/dl; absolute neutrophil count (ANC) ≥ 1.5 × 10^9^/L; platelet count ≥ 100 × 10^9^/L; serum bilirubin ≤ 1.5 × upper limit of normal (ULN); alanine aminotransferase (ALT) and/or aspartate aminotransferase (AST) ≤ 2.5 × ULN; serum creatinine ≤ ULN or calculated creatinine clearance ≥ 60 ml/min for subjects with creatinine levels > 1.5 × ULN; albumin ≥ 25 g/L; and international normalised ratio (INR) or prothrombin time (PT) and activated partial thromboplastin time (APTT) ≤ 1.5 × ULN unless receiving anticoagulant therapy. All patients gave written informed consent in accordance with institutional guidelines before study treatment.

Exclusion criteria included prior radiotherapy to the pelvis, significant urinary incontinence or bladder instability, active autoimmune disease, known history of immunodeficiency, active hepatitis B or C and use of systemic corticosteroids within 7 days of the first dose of trial treatment. Intravesical BCG treatment was not permitted within 30 days prior to the first dose of pembrolizumab and prior chemotherapy within 2 weeks.

### 2.2 Study design

An open label, Phase 1, dose escalation, safety run‐in study (NCT03167151) was initiated across two centres in the United Kingdom, to establish the safety, tolerability and toxicity of intravesical administration of pembrolizumab after TURBT, prior to a planned parallel group, randomised Phase 2 marker lesion study. The recruitment target for the safety run‐in phase was six patients.

The primary outcome measures were dose limiting toxicity (DLT) and tolerability, with incidence and severity of adverse events (AEs) graded using NCI Common Terminology Criteria for Adverse Events (CTCAE) v4.03. DLTs were defined as a clinically significant, drug‐related, Grade 4 haematological or Grade 3 or higher non‐haematological toxicity occurring within 7 days of administration of the first treatment at a given dose for that patient. Administration of at least five out of six treatments was required for the regime to be defined as tolerable.

Patients were treated once weekly with intravesical pembrolizumab in three paired patient cohorts with intra‐ and inter‐patient dose escalation (Table 1). This was to enable rapid escalation to the maximum administered dose as toxicities were not anticipated to be dose dependent and to allow evaluation of tolerability of repeated dosing using as few patients as possible. A starting dose of 50 mg was selected to ensure no unexpected local adverse reactions as pembrolizumab had not previously been administered via the intravesical route. A maximum intravesical dose of 200 mg was chosen to ensure acceptable tolerability should systemic absorption occur, with the cumulative dose when given once weekly remaining below the 10 mg/kg level previously shown to be tolerated when given intravenously on a two‐weekly schedule. A schedule of weekly intravesical treatments over a period of 6 weeks was chosen consistent with the schedule for BCG‐based induction immunotherapy. Trial treatment was initiated 14 days after TURBT or as close to this date as possible. Treatment start dates were staggered between patients, and treatment of patients in Cohorts 2 and 3 did not commence until both patients in the preceding cohort had cleared the DLT period for the D15 dose. If more than one patient experienced a DLT at a certain dose, this dose would be declared non‐tolerated and further escalation would cease.

**TABLE 1:** Dose escalation scheme

Cohort	Patients	Dose intravesical pembrolizumab (mg)		
		Day 1 + 8	Day 15 + 22	Day 29 + 36
1	1 + 2	50	100	200
2	3 + 4	100	200	200
3	5 + 6	200	200	200

Pembrolizumab was instilled as a 40 ml infusion into the bladder via a urinary catheter over a period of no more than 5 min and the patient instructed to retain the instilled suspension in the bladder for 2 h. Safety and tolerability assessments, blood tests including liver function and urinalysis were performed at each scheduled study visit and at the end of treatment visit on Day 92. Thyroid function was checked monthly.

### 2.3 Pharmacokinetic analyses

Blood samples were taken pre‐dose and 2 h post‐dose for the first treatment administration and pre‐dose at Cycle 6 (Day 36, after administration of at least two 200 mg doses) for a limited pharmacokinetic analysis of the systemic absorption of pembrolizumab by enzyme‐linked immunosorbent assay (ELISA). Briefly, half area high protein‐binding 96‐well plates (Corning) were coated with 25 μl of 13 μg/ml recombinant PD‐1 (a gift from Prof S Davis, University of Oxford) at 4°C overnight. After 5× phosphate‐buffered saline (PBS)/tween and 5× PBS washes to remove excess recombinant PD‐1, 100 μl of 10% fetal calf serum (FCS) PBS (blocking buffer) was added to the plate at 37°C for 2 h to avoid non‐specific binding of serum antibodies. Serum previously collected from melanoma patients treated with intravenous pembrolizumab was used as a positive control. Serial dilutions of serum in blocking buffer were then added and incubated at 4°C overnight. Plates were washed 5× with PBS/tween and 5× with PBS, before addition of 25 μl of anti‐human IgG‐horseradish peroxidase (HRP) in blocking buffer and incubation for 2 h at room temperature. After 10× PBS/tween washes, 25 μl of substrate was added and the reaction terminated on addition of 25 μl of stopping solution (2 N sulfuric acid). The optical densities of the wells were determined by reading the plate in an absorbance spectrophotometer at wavelengths of 490 nm.

### 2.4 Pharmacodynamic analyses

Samples of tumour and biopsies of normal bladder tissue, bladder barbotage and blood samples for immunoprofiling were obtained at the time of TURBT. Further blood samples for immunomonitoring were obtained before each treatment administration.

Tumours and normal biopsies were dissociated mechanically and enzymatically using a tumour dissociation kit (Miltenyi Biotec 130‐095‐929). Samples were washed with PBS and incubated with Aqua Fixable viability dye (ThermoFisher Scientific) for 10 min at room temperature in the dark. Next, cells were washed with PBS supplemented with 10% fetal bovine serum (FBS). Cells were resuspended in Brilliant Stain buffer (BD) with fluorescently labelled antibodies against CD4 (BV711, BD Bioscience 563033), TCR‐αβ (FITC, BD Bioscience, 561673), HLA‐DR (APC/Cy7, Biolegend 307617), CD8 (PerCP/Cy5.5, Biolegend 344709), CD38 (PE/Dazzle 594, Biolegend 356629), CD279 (PD‐1; APC, Biolegend 367405), CD326 (EpCAM; BV421, BD 563180), IgG4 Fc (PE, Cambridge Bioscience Ltd 9200‐09) and CD45 (PE‐Cy7, Biolegend 304015) and incubated for 20 min at 4°C in the dark. Cells were washed twice with PBS supplemented with 10% FBS. Cells were acquired on a BD FACSAria Fusion cell sorter. All flow cytometry data were analysed using FlowJo software v10 (TreeStar Inc.).

Live frozen peripheral blood mononuclear cells (PBMCs) were thawed and washed with Roswell Park Memorial Institute (RPMI) supplemented with 10% FBS and 12.5 U/ml Benzonase (Merck). Two million PBMCs of each sample were washed with PBS and incubated with Aqua Fixable viability dye (ThermoFisher Scientific) for 10 min at room temperature in the dark. Next, cells were washed with PBS supplemented with 5% FBS, 2 mM ethylenediaminetetraacetic acid (EDTA). Cells were resuspended in Brilliant Stain buffer (BD) with fluorescently labelled antibodies against CD8 (BUV395, BD 563795), CD16 (BUV496, BD, 612914), CD56 (BUV563, BD 612928), CD4 (BUV805, BD 564911), CXCR3 (BV421, Biolegend 353715), CCR4 (BV605, Biolegend 359417), CD3 (BV650, Biolegend 563999), CD45RA (BV711, Biolegend 304137), HLA DR (BV785, Biolegend 307642), CD25 (BB515, BD 564468), TCR‐γδ (BB700, BD 745944), CD38 (BB790‐P, BD 624296), PD‐1 (PE‐eFluor 610 Invitrogen), CD127 (PE, Beckman Coulter), ICOS (APC, Biolegend 313509), CD27 (Alexa Fluor 700, Biolegend 302813) and CCR7 (APC/cyanine, Biolegend 353212) and incubated for 20 min at room temperature in the dark. Cells were washed twice with PBS supplemented with 5% FBS, 2 mM EDTA. Cells were acquired on a BD LSR Fortessa X50 Flow Cytometer. All flow cytometry data were analysed using FlowJo software v10 (TreeStar Inc.). Clustering of downsampled gated CD3^+^ T cell populations was performed using the FlowJo plugin FlowSOM.

### 2.5 Statistical analyses

For patient baseline characteristics, numbers (with percentages) for binary and categorical variables and means (and standard deviations), or medians (minimum, maximum) for continuous variables are presented. The number of patients experiencing DLTs per cohort and AEs tabulated by cohort is reported. No formal comparison between dose levels was undertaken. Analyses were performed using Stata Version 15.1 (StataCorp, College Station, TX, USA). For exploratory analysis of the mean ± SEM expression of PD‐1 on T cells, a two‐tailed paired *t* test was performed using GraphPad PRISM Version 9.2.0.

This study has been conducted as part of the portfolio of trials in the registered UK Clinical Research Collaboration (UKCRC) Oxford Clinical Trials Research Unit (OCTRU) at the University of Oxford. It has followed their Standard Operating Procedures ensuring compliance with the principles of Good Clinical Practice and the Declaration of Helsinki and any applicable regulatory requirements. It was sponsored by the University of Oxford and approved by relevant regulatory and independent ethics committees.

## 3 RESULTS

### 3.1 Patient characteristics

**FIGURE 1:**
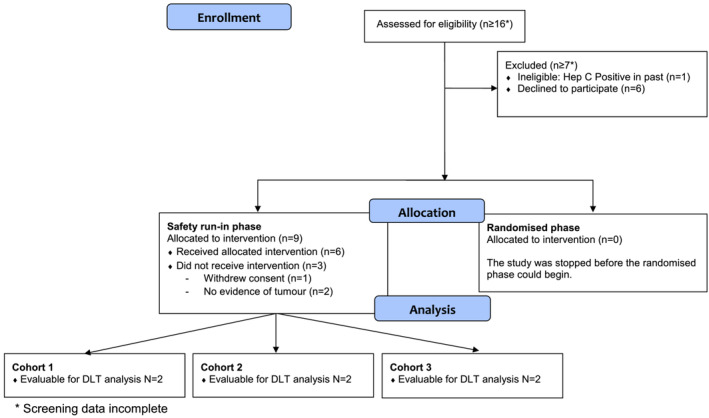
CONSORT flow diagram. DLT, dose limiting toxicity

Nine patients were recruited from one centre, of which six started treatment as allocated (Figure 1). Of the three patients who did not start treatment, two were withdrawn as they did not meet eligibility criteria and one patient withdrew their consent. Baseline characteristics of the six patients treated are listed in Table 2.

**TABLE 2:** Baseline characteristics

Cohort Day 1 starting dose	Safety Cohort 1 50 mg (*n* = 2)	Safety Cohort 2 100 mg (*n* = 2)	Safety Cohort 3 200 mg (*n* = 2)	Total (*n* = 6)
**Demographic**				
Age (years), mean (SD)	73 (0.0)	66 (10.6)	81 (7.8)	73 (8.9)
Gender				
Female, *n* (%)	0	1 (50%)	0	1 (16.7%)
Male, *n* (%)	2 (100%)	1 (50%)	2 (100%)	5 (83.3%)
Ethnicity				
White British, *n* (%)	2 (100%)	2 (100%)	2 (100%)	6 (100%)
Smoking status				
Current, *n* (%)	0	1 (50%)	0	1 (16.7%)
Ex‐smoker, *n* (%)	2 (100%)	1 (50%)	1 (50%)	4 (66.7%)
Never smoked, *n* (%)	0	0	1 (50%)	1 (16.7%)
ECOG Performance Status				
0, *n* (%)	2 (100%)	2 (100%)	2 (100%)	6 (100%)
1, *n* (%)	0	0	0	0
Prior intravesical treatment				
Yes, *n* (%)	2 (100%)	2 (100%)	2 (100%)	6 (100%)
Prior treatment: BCG	0	1 (50%)	1 (50%)	2 (33.3%)
Prior treatment: Mitomycin C	2 (100%)	2 (100%)	2 (100%)	6 (100%)
No, *n* (%)	0	0	0	0

Abbreviations: BCG, Bacillus Calmette‐Guerin; ECOG, Eastern Cooperative Oncology Group.

### 3.2 Dose escalation, administration and safety

Dose escalation proceeded as planned according to the schedule in Table 1 with no DLTs observed. Twenty‐two AEs were seen with five of the six patients (83%) experiencing one or more AEs. Fourteen were considered to be treatment related. One serious AE (urosepsis) was reported and was considered unlikely (probably not) related to pembrolizumab. All AEs, regardless of causality, are reported in Table 3. There were no Grade 3 or higher AEs related to pembrolizumab. All patients who started treatment completed six doses of treatment at their allocated doses, leading to the treatment regime being defined as tolerable.

**TABLE 3:** Adverse events regardless of causality, split by cohort and CTCAE grade

Cohort Day 1 starting dose	Safety Cohort 1 50 mg (*n* = 2)			Safety Cohort 2 100 mg (*n* = 2)		Safety Cohort 3 200 mg (*n* = 2)		Total (*n* = 6)
	CTCAE G1	CTCAE G2	CTCAE G3	CTCAE G1	CTCAE G2	CTCAE G1	CTCAE G2	
**AE lower level term**								
Cystitis	1	0	0	0	0	0	0	1
Dizziness	0	0	0	0	0	1	0	1
Dry cough	0	0	0	0	0	1	0	1
Dysuria	1	0	0	0	0	1	0	2
Fatigue	1	0	0	0	0	1	0	2
Fever	1	0	0	0	0	0	0	1
Haematuria	0	0	0	0	0	1	0	1
Hot flushes	0	0	0	0	0	1	1	2
Itching	0	0	0	0	0	1	0	1
Nausea	0	0	0	1	0	0	0	1
Rash over bilateral shins	0	0	0	0	0	1	0	1
Rigors	1	0	0	0	0	0	0	1
Urinary frequency and urgency	0	0	0	0	0	0	1	1
Urgency–frequency syndrome	0	0	0	0	0	2	0	2
Urinary tract infection	1	1	0	0	0	1	0	3
Urosepsis	0	0	1	0	0	0	0	1
**Total**	6	1	1	1	0	11	2	22

Abbreviations: AE, adverse event; CTCAE, Common Terminology Criteria for Adverse Events; G, Grade.

### 3.3 Pharmacokinetic analyses

**FIGURE 2:**
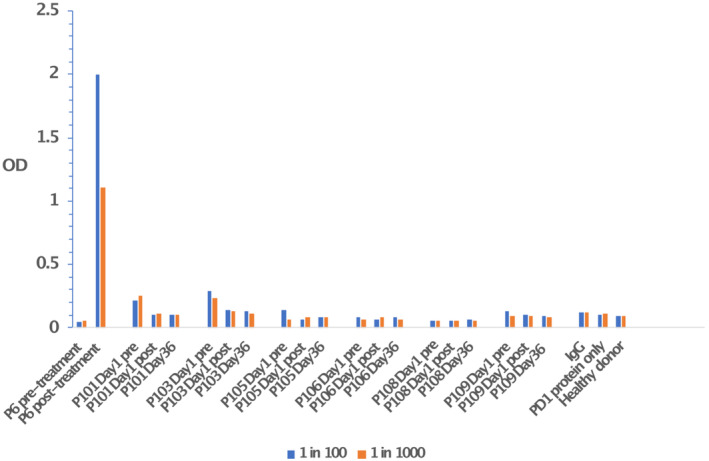
Level of pembrolizumab in serum before and after treatment. Serum from the indicated patients (P101–109) at the indicated timepoints was tested in ELISA for binding to soluble human PD‐1. Each serum was tested at 1:100 and 1:1000 dilutions; negative controls included recombinant immunoglobulins (IgG); PD‐1 protein alone and serum from healthy volunteers. Patient P6 is a melanoma patient treated intravenously with pembrolizumab and the serum was used as positive control. OD, absorption at 490 nm

Pembrolizumab was not detectable in the serum of any of the treated patients by ELISA either 2 h after the first intravesical dose of pembrolizumab or before the sixth dose on D36 (Figure 2). By contrast, pembrolizumab was detectable at dilutions of between 1:100 and 1:1000 in the positive control of serum from a melanoma patient 3 weeks post administration of intravenous pembrolizumab.

### 3.4 Pharmacodynamic analyses

**FIGURE 3:**
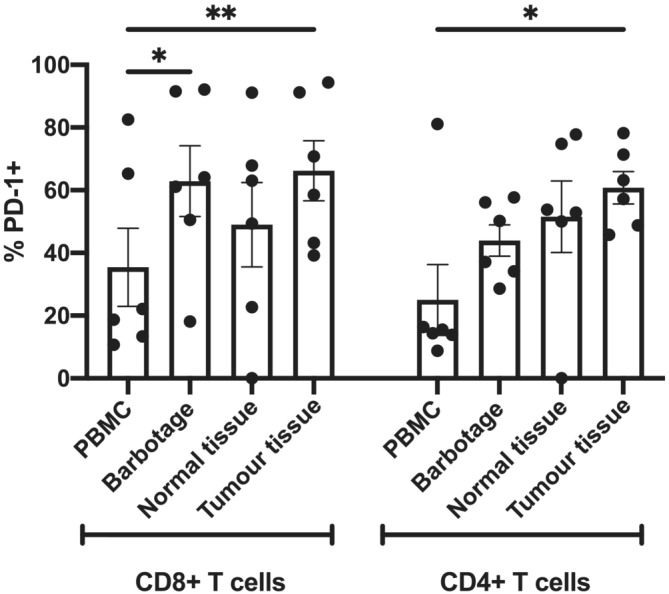
Evaluation of PD‐1 expression on T cells. Mean ± SEM expression of PD‐1 at baseline on CD8^+^ and CD4^+^ T cells freshly isolated from peripheral blood, bladder barbotage, normal bladder tissue and tumour tissue. PBMC, peripheral blood mononuclear cell. *p < 0.05, **p < 0.01, paired *t* test

The immune composition of fresh tumour samples obtained from patients at the time of TURBT was compared with that of matched peripheral blood, normal bladder tissue and bladder barbotage samples by flow cytometry (Figure S1). Expression of PD‐1 on both CD8^+^ and CD4^+^ T cells isolated from tumour tissue but not surrounding normal bladder tissue was significantly higher than on T cells isolated from the peripheral blood (Figure 3). PD‐1 expression on CD8^+^ but not CD4^+^ T cells obtained from bladder barbotage was also elevated compared with peripheral blood.

Immune monitoring by flow cytometry of PBMCs on a weekly basis throughout treatment showed no consistent alterations in the composition or phenotype of different T cell populations in response to intravesical pembrolizumab administration (Figure S2a–c). No changes in expression of PD‐1 were seen on CD4^+^ or CD8^+^ T cells over the course of treatment (Figure S3).

## 4 DISCUSSION

Recent advances in the treatment of patients with metastatic and high‐risk NMIBC following the success of anti‐PD‐1 checkpoint inhibitors have raised the possibility of targeting the PD‐1/PD‐L1 axis for treatment in lower risk NMIBC. Although anti‐PD‐1 therapies are generally well tolerated, systemic side effects that include immune‐related toxicities affecting the endocrine, gastrointestinal and respiratory systems can result in long‐term complications. Intravesical drug delivery offers the potential to reduce the systemic side effects and emerging pre‐clinical data suggest that intravesical administration of pembrolizumab is efficacious in orthotopic mouse models for treatment of localised urothelial bladder cancer.^22^

Six patients were successfully treated during this safety run‐in Phase 1 study, and treatment appears to be well tolerated. No DLTs were seen, and most patients experienced only low‐grade toxicities. Many of the AEs observed, such as dysuria and urgency–frequency syndrome, are commonly seen with other treatments delivered via the intravesical route so could be related to the route of administration of the pembrolizumab rather than the drug itself. No confirmed systemic immune‐related toxicities were seen. All patients were able to complete the planned number of treatments, suggesting that this regime of intravesical pembrolizumab administration is tolerable.

Analysis of the immune infiltrate present in the tumour at the time of TURBT prior to treatment confirmed higher PD‐1 expression on tumour infiltrating lymphocytes relative to those in the periphery, suggesting a degree of pre‐existing immune cell exhaustion and supporting targeting PD‐1/PD‐L1 interactions as a way to augment anti‐tumour responses. However, interestingly, expression on immune cells recovered from normal tissue and from bladder barbotage was also elevated, perhaps suggesting a degree of local inflammation.

Administration of intravenous anti‐PD‐1 therapy is typically accompanied by proliferation and activation of PD‐1^+^ peripheral T cell populations.^23^, ^24^ In addition, binding of the anti‐PD‐1 treatment to PD‐1 on the surface of T cells typically results in abrogation of direct staining of PD‐1 by antibodies used for flow cytometry in treated patients.^25^, ^26^ Monitoring of peripheral immune cell populations in patients over the course of this study by flow cytometry revealed no consistent changes in the composition or phenotype of any peripheral immune subsets in response to weekly administration of intravesical pembrolizumab. Furthermore, no changes in either the abundance or intensity of staining of PD‐1^+^ CD8^+^ or CD4^+^ T cell populations were seen over the course of intravesical pembrolizumab administration. Consistent with this, pharmacokinetic analyses did not identify any detectable pembrolizumab in the serum of patients at either 2 h after administration of the first dose or at the sixth week of treatment, suggesting either that pembrolizumab is not systemically absorbed following intravesical administration at the doses and schedule used in this study, or that this is at levels below the limit of detection of our ELISA and below levels reported to cause diminished PD‐1 staining. Taken together, the data from this study would suggest that intravesical administration of pembrolizumab does not appear to have either direct or indirect effects upon the systemic immune response. Although this may be advantageous in terms of potentially minimising immune‐related toxicities, it remains to be determined whether intravesical administration of anti‐PD‐1 antibody does indeed cause blockade of PD‐1/PD‐L1 interactions intra‐tumourally and whether this is able to augment a local anti‐tumour immune response in the absence of a systemic immune response. Further studies will be required to address this and establish whether this translates to clinical benefit.

A randomised, parallel group, Phase 2 marker lesion study of intravesical or intravenous pembrolizumab was planned following this safety run‐in to provide a further assessment of safety and tolerability as well as a preliminary assessment of efficacy and intra‐tumoural effects of pembrolizumab in intermediate‐risk recurrent NMIBC. However, due to delays in recruitment to the safety run‐in, the decision was taken not to proceed with this part of the study. As such, a significant limitation of this study is that the dose of pembrolizumab that is active intravesically has not been established. Ongoing studies including NCT02808143 looking at intravesical pembrolizumab in combination with intravesical BCG may provide further information in this regard. Although the maximum dose of 200 mg administered in this study was well tolerated and is consistent with doses used in systemic administration in other settings, it is not known whether a dose of 200 mg would be required for any potential therapeutic effect when administered via the intravesical route. The choice of dose for further study in Phase 2 trials could therefore be guided by evidence of on‐target pharmacodynamic effects and pragmatic considerations including cost.

In summary, administration of intravesical pembrolizumab was well tolerated and did not raise any safety concerns in patients with NMIBC following TURBT. There was no evidence of systemic absorption or systemic immune effects following intravesical administration. Further studies are required to assess whether intravesical administration has anti‐tumour activity.

## DISCLOSURE OF INTEREST

The authors declare the following competing interests:

- V.K.W. has received support for conference attendance and travel from MSD.
- M.S. is an employee of Immunocore and a consultant for Nucleome.
- M.R.M. has received grants from Roche, grants from AstraZeneca, grants and personal fees from GSK, personal fees and other from Novartis, other from Millenium, personal fees and other from Immunocore, personal fees and other from BMS, personal fees and other from Eisai, other from Pfizer, personal fees, non‐financial support and other from Merck/MSD, personal fees and other from Rigontec (acquired by MSD), other from Regeneron, personal fees and other from BiolineRx, personal fees and other from Array Biopharma (now Pfizer), non‐financial support and other from Replimune, personal fees from Kineta and personal fees from Silicon Therapeutics, outside the submitted work.
- J.‐L.C., K.P., C.B., L.C., C.H., G.V., L.E., C.R., A.T., M.R., G.N., J.C. and A.S.P. declare no competing interests.

## AUTHOR CONTRIBUTIONS

V.K.W., G.N., M.S., M.R.M., V.C., J.C. and A.S.P.: Conceptualisation. V.K.W., J.‐L.C., L.E., C.R., A.T., M.R., G.N., M.S., M.R.M., V.C., J.C. and A.S.P.: Methodology. J.‐L.C., L.E., C.R. and M.S.: Formal analysis. J.‐L.C., K.P., C.H., G.V., A.T., J.C. and A.S.P.: Investigation. J.‐L.C., K.P., C.H., G.V., A.T., M.R., M.S., M.R.M., V.C., J.C. and A.S.P.: Resources. V.K.W., J.‐L.C. and K.P.: Writing—original draft. V.K.W., J.‐L.C., K.P., C.B., L.C., C.H., G.V., L.E., C.R., A.T., M.R., G.N., M.S., M.R.M., J.C. and A.S.P.: Writing—review and editing. V.K.W., J.‐L.C., L.E., C.R. and M.S.: Visualisation. J.C., A.S.P., V.C. and M.R.M.: Supervision. L.C. and C.B.: Project administration. V.K.W., M.R.M., V.C., J.C. and A.S.P.: Funding acquisition.

## Supporting information


**Figure S1.** Gating strategy for FACS analysis of PD‐1 expression on T cells freshly isolated from peripheral blood, bladder barbotage, normal bladder tissue and tumour tissue
**Figure S2.** Immune monitoring by flow cytometry of T cell populations in peripheral blood over the course of pembrolizumab administration.
**Figure S3:** PD1 expression on peripheral CD8+ and CD4+ T cells over time. Analysed by gating strategy shown in Supplementary Figure 2a.
