# Supplementary material for: PemBla: A Phase 1 study of intravesical pembrolizumab in recurrent non‐muscle‐invasive bladder cancer
Source: BJUI Compass. 2023 Jan 13;4(3):322–30. doi: 10.1002/bco2.220 (PMC10071078; doi:10.1002/bco2.220)
Supplement: Supplementary file 1 — Figure S1. Gating strategy for FACS analysis of PD‐1 expression on T cells freshly isolated from peripheral blood, bladder barbotage, normal bladder tissue and tumour tissue Figure S2. Immune monitoring by flow cytometry of T cell populations in peripheral blood over the course of pembrolizumab administration. Figure S3: PD1 expression on peripheral CD8+ and CD4+ T cells over time. Analysed by gating strategy shown in Supplementary Figure 2a. [file BCO2-4-322-s001.docx]

**Supplementary Figures**

**Supplementary Fig. 1**. Gating strategy for FACS analysis of PD-1 expression on T cells freshly isolated from peripheral blood, bladder barbotage, normal bladder tissue and tumour tissue

**Supplementary Figure 2.** Immune monitoring by flow cytometry of T cell populations in peripheral blood over the course of pembrolizumab administration.

2a. Gating strategy for ex-vivo FACS analysis of peripheral blood mononuclear cells

2b: Heatmap showing median fluorescent marker intensities of T cell populations identified by FlowSOM clustering of manually gated CD3+ T cells

2c. Frequency of CD3+ T cell subsets per patient in the peripheral blood identified by FlowSOM clustering over time. CM, central memory (CD45RAneg, CCR7pos); TEMRA: Terminally differentiated effector memory T cells (CD45RApos, CCR7neg). Activated cells are defined as CD38pos and HLADRpos.

**Supplementary Figure 3:** PD1 expression on peripheral CD8+ and CD4+ T cells over time. Analysed by gating strategy shown in Supplementary Fig. 2a**.**
